# Definitional ambiguity in cognitive warfare: a critical and systematic conceptual review through ideal-type analysis

**DOI:** 10.3389/fdata.2026.1762571

**Published:** 2026-05-15

**Authors:** Per-Erik Nilsson, Andreas Haga, Kristina Hellström

**Affiliations:** Department of Defense Analysis, Swedish Defense Research Agency (FOI), Stockholm, Sweden

**Keywords:** cognitive warfare, information technology, information warfare, propaganda, psychological warfare, strategic communications

## Abstract

Cognitive warfare is a relatively new concept in both military and academic discourse. The article's purpose is to advance conceptual clarity regarding cognitive warfare and to support future policy-oriented and academic research that strengthens the field's conceptual and methodological foundations, understood here as the broader domain of communication and defense studies concerned with informational and cognitive forms of contestation. This article examines how the notion is conceptualized within the emerging body of research, drawing on a systematic literature review. With support from LLM-assisted analysis, the study employs an exploratory methodology to identify both conceptual commonalities and points of divergence. The review indicates that cognitive warfare remains an underdeveloped research field, characterized by broad assumptions and limited scientific rigor. While the concept may represent a reframing of long-standing practices, it may also serve a political function by drawing renewed attention to forms of influence and conflict that have been overshadowed in recent decades. The article concludes by outlining avenues for future interdisciplinary research, emphasizing the need for conceptual clarity, empirical operationalization, and a more nuanced understanding of how adversaries themselves articulate and employ cognitive warfare.

## 1 Introduction

Cognitive warfare is a relatively new concept in both military and academic discourse. As it gradually makes inroads into current thinking, it is clear that several views of what the concept means are being discussed. This raises the question: What is cognitive warfare, and what does it contribute to better understanding the ever-evolving contours of warfare? In the research literature, the first explicit use of the term cognitive warfare is typically attributed to a thesis by Dahl (1996) at the School of Airpower Studies at the United States Air University in Alabama. In the thesis, Dahl presents cognitive warfare as an “analytical tool” within the framework of command and control warfare (C2W), explained as the integrated use of psychological, electronic, deceptive, and physical operations, supported by intelligence (2). Dahl analyses 20th-century wars to understand the stressors that hamper decision-making processes, arguing that cognitive warfare

“compels one to understand the target command prior to applying stress and deception schemes against it.” (130)

At the time of publication, a bundle of other concepts was used in military and academic writing to capture the new developments of warfare: compound warfare, netcentric warfare, and information warfare, to name a few. Cognitive warfare, however, did not gain traction. However, during the American Department of Defense Intelligence Information System (DoDIIS) World Wide conference in 2017, the Director of the Defense Intelligence Agency, Lt. Gen. Stewart (2017), gave a one-hour-long speech on the development of warfare. After explaining the first through fourth generations of warfare, Lt. Gen. Stewart argues, “Fifth Generation Warfare, it will be Cognitive Warfare.” He continues to explain that in the “21^st^ century, warfare is about winning the information and the decision space, either before or during a conflict,” while adding: “This is the decisive factor.” For Lt. Gen. Stewart, cognitive warfare appears to be a development of age-old warfighting, going back to the Chinese military theorist Sun Tzu's over two-millennia-old theories on “breaking the enemy's resistance without fighting.” The novelty of cognitive warfare is understood to reside in the explicit mixture of maneuver warfare coupled with information and cyber-attacks to shape the battlefield to manipulate and exploit the adversary's perception of the order of things:

“You might not even think you're at war, but your adversaries are busy shaping the battlefield and undermining the order you seek to uphold.”

In 2020, the North Atlantic Treaty Organization's (NATO) Allied Command Transformation (ACT) initiated analytical work on cognitive warfare, followed by research initiatives within NATO's Science and Technology Organization. In a public statement from NATO ACT (2024), it was revealed that a Cognitive Warfare Concept was under development to face “a new kind of threat,” described as “a war fought not with bombs and missiles, but with lies and manipulation… by spreading disinformation and confrontational rhetoric online… aiming to sow discord and weaken our ability to protect ourselves effectively.”

These approaches to cognitive warfare are not mutually exclusive. Still, they differ in emphasis: shifting from an analytical framework for understanding 20th-century conflicts, to highlighting modern warfare as the effort to deprive adversaries of the ability to think and act, and finally, to framing it as a new form of threat rooted in deception and manipulation. This again raises the question: What is cognitive warfare, and what does it contribute to better understanding the ever-evolving contours of warfare?

Academic research on war and warfare serves a variety of purposes across a broad range of disciplines. To name a few, academic inquiry may focus not only on understanding war as a human and historical phenomenon (Hoffenaar, 2021; Keegan, 1991; Keeley, 1996); and on ethics, legality, and public oversight (Soeters et al., 2014; Walzer, 2004; Zehfuss, 2018); but also on advancing military strategy and technology (Benfield and Greg, 2021; Meyer et al., 2019; Newlove-Eriksson and Eriksson, 2023). Moreover, academic research on military development raises a range of ethical and legal issues (Emery, 2021; Pawiński, 2018).

Against this background, the article's purpose is to advance conceptual clarity regarding cognitive warfare and to support future research that strengthens the field's conceptual and methodological foundations, domain of communication and defense studies concerned with informational and cognitive forms of contestation. The specific aim is to conduct a critical systematic literature review using an exploratory methodology that combines qualitative review methods with quantitative large language model (LLM)-assisted analysis.

To achieve the purpose and the aim, the article sets out to understand how cognitive warfare is conceptualized in the literature by answering six research questions (RQs):

RQ1: What are the main contours of the research field on cognitive warfare?
RQ2: What is cognitive warfare (i.e., its definition)?
RQ3: Why is cognitive warfare practiced (i.e., its purpose)?
RQ4: How is cognitive warfare operationalized (i.e., its components and methods)?
RQ5: Where is cognitive warfare situated in relation to other types of warfare (i.e., its distinctions)?
RQ6: By assessing the answers to RQ1–5, what future avenues of research are needed to enhance the conceptual clarity of cognitive warfare?

This article does not assume a single unified audience for the concept of cognitive warfare. Instead, it treats conceptual clarity as dependent on purpose and context. As discussed in Section 2.2, clarity in academic research supports comparison, cumulative knowledge, and operationalization. In policy and military practice, by contrast, concepts are more often judged by their usability, flexibility, and ability to guide action under uncertainty. This study therefore assesses cognitive warfare primarily as an analytical category within scholarly research, focusing on definitional coherence, differentiation, and analytical utility, while recognizing that conceptual ambiguity may still allow for institutional usefulness. This distinction matters because a concept can be practically useful without being analytically precise. The aim of this article is not to judge the institutional usefulness of cognitive warfare, but to clarify its meaning and assess its adequacy for scholarly analysis.

The article proceeds as follows. The next section reviews prior literature on cognitive warfare and outlines key preconditions for both policy-oriented and academic research. Section 3 sets out the research design and methodology. The review findings are then presented according to RQ1–RQ5, with RQ6 addressed in the subsequent section. The article ends with a brief conclusion.

## 2 Background

### 2.1 Previous reviews

The most rigorous review of the concept of cognitive warfare is Deppe and Schaal's (2024) conceptual analysis of NATO ACT's exploratory concept. They set out to reduce conceptual ambiguity between military and academic understandings of cognitive warfare and to advance its development across both domains. Their study evaluates the concept using Gerring's (2001) eight criteria of conceptual goodness (coherence, operationalization, validity, field utility, resonance, contextual range, parsimony, and analytical or empirical utility).^1^ They note that while the NATO concept was devised to fulfill a specific doctrinal function, its empirical validation is hindered by a lack of real-world cases. The concept displays high internal coherence but lacks sufficient external differentiation from related notions such as hybrid threats and hybrid warfare, raising the question of whether it should be treated as a subordinate or an integrative concept within those frameworks. Although its extensive attributes and technological focus complicate operationalization, its forward-looking orientation aligns with its purpose as a military concept. Deppe and Schaal propose a streamlined, interoperable redefinition centered on cognitive effects, warfare elements, and technology, to enhance analytical precision and empirical applicability while retaining alignment with NATO ACT's broader framework.

Another review by Ibrahim et al. (2023) compares cognitive warfare and psychological warfare to determine whether the former constitutes an evolutionary successor to the latter. They conduct a systematic review using Lasswell's (1948) communication model as an analytical framework; they find substantial definitional overlap, suggesting that cognitive warfare represents a modernized continuation of psychological warfare, shaped by new technologies such as social media. The authors argue that psychological operations and information warfare can be understood as subcomponents or practical expressions within the broader framework of cognitive warfare.

The most recent review was conducted by Muñoz Plaza et al. (2023). While the subtitle of this conference paper is “A Systematic Review,” it contains no discussion and delimitation of the selected sources for the review, nor any discussion of methodology.

The low number of review articles in the field indicates that it is relatively immature, as this article also shows. Nonetheless, this article builds on these earlier studies by taking a broader analytical step back to map the research field and examine the conceptual heterogeneity surrounding cognitive warfare.

### 2.2 Academic and military concepts

The humanities and social sciences engage with theoretical concepts that often have one or several commonsensical meanings. Concepts such as politics, democracy, economics, culture, religion, and art guide academic disciplines as objects of inquiry, but the dissonance between commonsensical and academic understandings can be substantial, as can the dissonance within different academic schools of thought in their ontological, epistemological, and normative foundations. In this sense, such terms exemplify what Gallie (1956) called essentially contested concepts,

“concepts the proper use of which inevitably involves endless disputes about their proper uses.” (169)

While Gallie emphasized the inherent contestation of such terms, later scholars (e.g., Collier et al., 2006) have systematized his insight into analytical frameworks to achieve conceptual coherence. The review by Deppe and Schaal (2024) is one such example. These approaches are valuable when the aim is conceptual cohesion. Still, since the purpose of this article is conceptual clarity, that is, making conceptual heterogeneity explicit, it is necessary to contrast how concepts function in academic inquiry and in military practice.

A military concept is typically presented as a forward-looking framework that articulates how armed forces might operate in anticipated future conditions, enabling experimentation, innovation, and adaptation to emerging challenges. Such concepts provide a vision rather than a prescription, identifying potential approaches, capabilities, and organizational changes needed to meet evolving strategic and operational demands (Benfield and Greg, 2021; NATO ACT, 2021; Pikner et al., 2012). While concepts project desired or possible futures, military doctrine ideally codifies the present by formalizing capabilities, structures, and operational methods (Meshkin, 2025; Smith, 2018).

In this regard, the scientific coherence of military concepts is less important than their functional utility, that is, how effectively they address operational demands within the military context in which they are applied. Whether cognitive warfare represents a genuinely new phenomenon or is narrowly confined to cognition is therefore secondary, provided the concept serves to guide future approaches, capabilities, and organizational development. Nevertheless, military concepts are not immune to academic borrowing and conceptual inflation, particularly when shaped by bureaucratic or political imperatives (Echevarria II, 2017). The adoption of the term *hybrid* in military and academic discourse is a case in point.

In an interview study of the pros and cons of the concept within NATO circles, Caliskan and Michel (2021) argue that while the concept of “hybrid warfare” has gained prominence since 2014, it has often obscured strategic understanding due to its vagueness and limited analytical value; nonetheless, it has been helpful as a political and bureaucratic tool to raise awareness, drive capability development, and secure resources.

To conclude, when approaching cognitive warfare as a military concept, its coherence and value should be assessed relative to the organizational and epistemic context in which it is employed. A key challenge for policy-oriented military research lies in working within conceptual frameworks already institutionalized in the organizations expected to apply its findings. Whereas academic research can treat concepts as essentially contested and open to continual redefinition, in a military setting, such rearticulation may generate ambiguity rather than clarity, undermining both communication and implementation. The extent to which conceptual clarification should prioritize analytical precision or institutional usability ultimately depends on the research's purpose. For purely academic or deconstructive inquiries, pursuing conceptual rigor and internal coherence may be entirely appropriate. For policy-oriented or applied studies, however, the value of a concept such as cognitive warfare lies less in theoretical purity than in its capacity to guide adaptation, capability development, and strategic practice within the institutional setting it serves.

## 3 Materials and methods

This article presents a critical systematic literature review employing an exploratory mixed methods approach. The systematic component involves the identification, critical appraisal, and synthesis of relevant research, using established research designs to produce transparent, structured reviews (Petticrew and Roberts, 2006; Snyder, 2019).

The exploratory mixed-methods dimension combines qualitative reading and coding of the selected literature with a semantic-similarity analysis to create an ideal-typical synthesis, supported by analytical assistance from OpenAI's LLM, ChatGPT-5. The review is considered critical in that it seeks to move beyond summarizing existing studies, instead engaging in the evaluation of limitations, conceptual gaps, and opportunities for future research (Grant and Booth, 2009). The subsequent sections elaborate on the epistemological foundation of the review, its design and sample selection, and the process for the ideal-typical synthesis.

### 3.1 Epistemological foundation

This literature review is based on a critical realist approach (Buch-Hansen and Nielsen, 2020). Critical realism holds that an objective reality exists independently of human perception, yet social, cultural, and temporal contexts always mediate human understanding of reality. In this view, knowledge is both fallible and situated: it reflects an ongoing human effort to apprehend a reality that is only partially accessible through our conceptual and perceptual frameworks.

In this article, the object of inquiry in the initial stages of the review (RQ1–RQ5) is the textual construction and representation of cognitive warfare, rather than the extent to which it constitutes an objectively real phenomenon. The effects denoted by the term are acknowledged as real, yet they would remain so even if labeled warfare type A, B, or C. A critical realist approach is therefore adopted in a pragmatic sense: it examines how a concept such as cognitive warfare is constructed, interpreted, and employed to meet specific epistemic and practical needs within both academic theorizing and military planning. In the final stage of the review (RQ6), the analysis turns normative, assessing how the current state of research addresses these needs and how conceptual and methodological refinements might advance both the academic and policy-oriented domains.

### 3.2 Review design and sample selection

The review design followed four steps (identification, screening, coding, and synthesis) and was facilitated by the review software Covidence. The initial identification of relevant literature was based on searches of academic research repositories (Scopus, Web of Science, and PubMed) conducted April 9–28, 2025, resulting in a sample of 1,211 sources. The search-based identification was complemented by iterative reference screening (“snowballing”), which added another 21 sources to the sample. Of the sample, 103 duplicates were removed, leaving 1,129 sources for screening.

The screening process was carried out in two parts. The first part involved reading abstracts, yielding 129 sources; the second part involved full-text reading of these sources, yielding a final corpus of 52 sources.

The keywords used for the initial search were “cognitive warfare,” and the selection criteria were academic articles in English explicitly employing the concept.^2^ Through snowballing, non-academic journal sources were included, since many of these were frequently referenced in the sample. These sources include reports from non-governmental organizations (NGOs) and think tanks, one academic book chapter, and reports and articles from NATO STO and ACT.

To qualitatively review the corpus, a coding scheme based on the research questions was used. It was developed iteratively with a sample of 10 sources from the corpus. The scheme was divided into five parts. The first part (RQ1) identified metadata of each source (year of publication, academic discipline, type of publication, contribution, thematic context, and key references). The second part (RQ2) focused on the *definitions* of cognitive warfare; the third part (RQ3) on the *purpose* of cognitive warfare; the fourth (RQ4) on the *components and methods* of cognitive warfare; and the final part (RQ5) on the *distinguishing* features of cognitive warfare vis-à-vis other types of warfare. Parts two to five involved manually selecting the relevant section in each source. The final product of the coding process was an extensive dataset.

Each author coded the material independently. The dataset was then jointly evaluated to create intersubjective coherence, including discussions about inconsistencies and diverging interpretations. Once agreement was reached, the dataset was reprocessed and prepared for automated ideal-typical synthesis with the assistance of the LLM.

### 3.3 Semantic similarity for ideal-typical synthesis

Previous research on the use of LLMs in research reviews has primarily been conducted within data science and has focused on model evaluation and development (Scherbakov et al., 2025). Examples include work on developing models for faster and more efficient summarization of large corpora (Kasanishi et al., 2023), the design of LLM-driven chatbots for querying research databases (Li et al., 2025), and comparisons between LLM-based and human-conducted literature reviews (Tang et al., 2025). Studies using LLMs and semantic similarity for literature review tasks have similarly emphasized efficiency and time reduction (Dhakal et al., 2025; Nguyen et al., 2025). No previous research was identified that either theoretically discusses or employs automated textual analysis in combination with qualitative coding for the purpose of conducting a literature review, although it is a standard procedure for automated analysis of empirical data (e.g., Benoit et al., 2019; Grimmer et al., 2022; Kozik et al., 2024).

The present study therefore adopts an exploratory methodological stance that draws on the Weberian tradition of ideal type analysis (Gerring, 2012; Stapley et al., 2022). Ideal types are simplified analytical constructs that highlight key features of a phenomenon in order to clarify conceptual variation and support comparison, rather than to mirror empirical reality in full. In this analysis, they are developed through a controlled synthesis of semantically similar definitions in the literature and used to trace variation in how cognitive warfare is defined.

The aim was to develop a procedure that captures the research field more fully than a purely thematic summary. The approach combines computational assistance with interpretive synthesis to produce a clearer and more reproducible account of the literature. More specifically, it brings together conceptual analysis (Collier and Mahon, 1993; Gerring, 1999) with the grouping of text excerpts from research questions two to five according to semantic similarity (i.e., shared meaning), following established practices in lexical semantics (Geeraerts, 2017) and qualitative content analysis (Krippendorff, 2019; Schreier, 2012).

The automated analytical process was thus used to complement the manual coding by identifying semantic similarity among the definitional excerpts using transformer-based embedding representations from the LLM (ChatGPT-5, 5 October 2025).^3^ The model grouped excerpts by proximity in meaning, and the resulting clusters were refined manually. Through iterative prompt engineering, the LLM was instructed to generate five ideal-typical syntheses for each research question, each representing a distinct conceptual cluster and ranked from most inclusive to most specific. The data processed by the LLM were the text excerpts from the sources. All outputs were manually reviewed, edited for conceptual accuracy, and validated through repeated cross-checking. Once the prompt was developed (Appendix 1), it was tested across three separate model accounts and benchmarked against Anthropic's LLM, Claude.

## 4 Results

In this section, the analysis of the conceptualization of cognitive warfare is described by answering the research questions:

RQ1: What are the main contours of the research field on cognitive warfare?
RQ2: How is cognitive warfare defined?
RQ3: What are the core components of cognitive warfare?
RQ4: What is the purpose of cognitive warfare?
RQ5: How is cognitive warfare distinguished from other, similar types of warfare?

Based on the corpus, these questions together address the broader contours of the research field of cognitive warfare, including its various definitions, internal structure, functional orientation, and external differentiation.

### 4.1 (RQ1) The contours of the field of research

**Figure 1:**
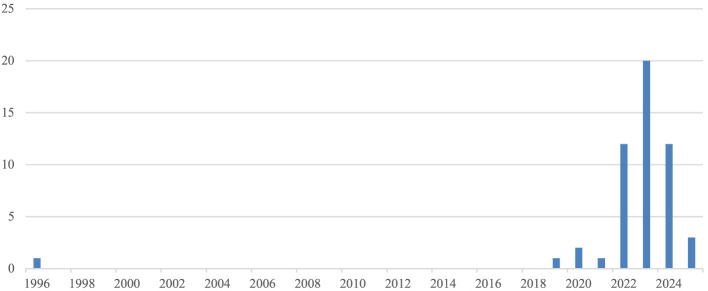
Number of corpus publications per year.

The years of publication in the review range from 1996 to 2025 (Figure 1). However, the publication in 1996 is the thesis mentioned above (Dahl, 1996), followed by publications in 2019 (n1), 2020 (n2), 2021 (1), 2022 (n12), 2023 (n20), 2024 (n12), and 2025 (n3).

Regarding the academic disciplines, the publications are, by and large, classified as belonging to the social sciences (n49), followed by health and welfare (n5), arts and humanities (n2), and information and communication technologies (n1).^4^ A handful (n7) of sources are a combination of two disciplines. Here, it must be noted that this dominance of the social sciences does not imply that research on cognitive warfare is lacking in general; rather, it indicates publications that explicitly use the concept.

The type-of-publication categories are: academic publications in journals listed in the Web of Science Journal Impact Factor (JIF) (n9); non-listed academic publications including preprints and one book chapter (n18); and “other” (n25), which includes public professional publications (e.g., NATO and NGO publications). This classification does not in itself make any claims about the academic quality of individual publications. However, it suggests that academic publications in non-academic outlets, such as NATO publications and NGO reports, currently dominate the literature on cognitive warfare.

The nature of contribution refers to whether the focus of the publications are primarily empirical (n3), conceptual (n44), or mixed (n5). The classification here has been deliberately generous toward the empirical category, including publications that engage with empirical material, whether or not the empirical work was conducted for that specific publication.

The thematic context aims to capture whether the publication is framed as theoretical and/or country- or organization-specific. “Theoretical” refers to a more thorough discussion of the concept of cognitive warfare than merely stating that cognitive warfare is X, Y or Z. One publication can fall into several of these categories: NATO (n27), Russia (n16), China (n10), Ukraine (n8), and theoretical (n10).

**Figure 2:**
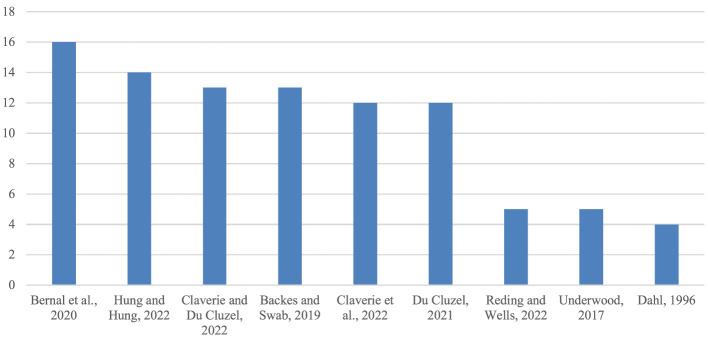
Most cited references in corpus.

Regarding key references (Figure 2), the presentation here is limited to publications that receive at least four references per article. The most frequently cited publication (n16) is a joint report by NATO and Johns Hopkins University (Bernal et al., 2020). In order of citation frequency, this report is followed by: (n14) an academic article published in a listed academic journal (Hung and Hung, 2022); (n13) a conference paper from a NATO ACT Innovation Hub conference in 2021 (Claverie and Du Cluzel, 2022); (n13) an NGO report (Backes and Swab, 2019), which is also the first publication in the corpus since 1996; (n12) a conference anthology from the above-mentioned NATO ACT conference (Claverie et al., 2022) which is not included in the corpus; (n12) a report from NATO ACT's Innovation Hub (Du Cluzel, 2021); (n5) a book chapter (Reding and Wells, 2022); (n5) a news piece (Underwood, 2017) reporting on the speech by Lt. Gen. Stewart cited in the introduction to this article, which is incorrectly referenced as an article on cognitive warfare itself but is, in fact, a short summary of the speech and therefore not included in the corpus; and, finally, (n4) the thesis mentioned above (Dahl, 1996), published in 1996.

Overall, cognitive warfare constitutes a young field of research that encompasses professional, conference, and preprint publications. Only a small number of academic publications appear in high-ranking, or even ranked, journals. The field is heavily dominated by conceptual and theoretical perspectives, with a marked lack of empirical references and clear operationalization. Finally, as a field, it is largely driven by professional and NATO STO publications.

### 4.2 (RQ2) Definitions of cognitive warfare

The ideal-type definitions of cognitive warfare are clustered according to the ways it is defined in each publication (see Table 1). The first and most inclusive (~85%) ideal-typical definition, “The Mind as a Battlespace,” stipulates that cognitive warfare targets human cognition in order to shape behavior and secure advantage in conflict or competition. This ideal type draws on statements that describe cognitive warfare as “mind control” (Drmotová and Kutěj, 2024), “the art of deceiving the brain” (Claverie, 2022), and “a multifaceted strategy that targets the human mind” (Meghraoui and Belkhamza, 2025).

**Table 1:** Definitions.

Cluster	Coverage	Title	Ideal-type
1	~85%	The mind as battlespace	Cognitive warfare deliberately targets human cognition—perceptions, beliefs, emotions, and decision-making—to shape behavior and secure advantage in conflict or competition.
2	~70%	Cognitive domain contestation	Cognitive warfare refers to a form of contestation that targets the cognitive domain by leveraging psychological, informational, and social processes.
3	~55%	Next-generation psychological and information warfare	Cognitive warfare evolves from psychological and information warfare, merging propaganda, disinformation, and strategic communication into a comprehensive contest for influence over human thought.
4	~40%	Techno-cognitive convergence	Cognitive warfare fuses emerging technologies within hybrid strategies to exploit vulnerabilities in human reasoning and collective cognition.
5	~25%	Covert subversive warfare	Cognitive warfare is a distinct and covert domain of conflict where the human mind becomes the primary terrain, expressed through attacks on knowledge, weaponized trust, and the corrosion of societal cohesion.

The second ideal-typical definition (~70%), “Cognitive Domain Contestation,” is similar but slightly more specific in identifying cognitive warfare as a particular domain of warfighting while highlighting psychological, informational, and social processes as vectors. Examples include seeing cognitive warfare as a “competition for the human mind and the ability to transform the worldview of people in society” (Danyk and Briggs, 2023) and “the crucial war domain that determines the outcome of modern wars” (Yun and Kim, 2022).

The third ideal-typical definition (~55%), “Next Generation Psychological and Information Warfare,” frames cognitive warfare as a development of “psychological and information warfare, merging propaganda, disinformation, and strategic communication into a comprehensive contest for influence over human thought.” Examples from the corpus include treating cognitive warfare as “a more expanded concept of psychological warfare” (Yun and Kim, 2022) and as “an extension of information warfare” (Danet, 2023).

The fourth ideal type (~40%), “Techno-Cognitive Convergence,” is more narrowly focused on “emerging technologies” such as AI, cyber operations, and social media, and is seen “within hybrid and gray-zone strategies to exploit vulnerabilities in human reasoning and collective cognition.” This includes defining cognitive warfare as “a tactic, which combines traditional and emerging technologies” (Deppe and Schaal, 2024) and “an integral part of hybrid warfare” (da Silva et al., 2025).

The final ideal-type definition (~25%), “Covert Subversive Warfare,” presents cognitive warfare as “a distinct and covert domain of conflict where the human mind becomes the primary terrain, expressed through attacks on knowledge, weaponized trust, and the corrosion of societal cohesion.” One example from the corpus defines cognitive warfare as “a war of ideologies that strives to erode the trust that underpins every society” (Du Cluzel, 2021).

To summarize, the most general definition of cognitive warfare is a behavior-centric form of warfare within the conceptual family of psychological and information warfare, encompassing propaganda and disinformation. It targets the human mind and the so-called cognitive domain, operating across the blurred boundaries of war and peace and extending beyond alleged traditional political or societal arenas. It is typically not limited to specific publics in theaters or operational environments, but to humanity at large. Drawing selectively on concepts from cognitive psychology (e.g., perception, emotion, and decision-making), it connects to both technology and religious, political, and social dynamics. The scientific concepts used are often grounded in neither deeper theoretical nor empirical foundations. Finally, at the time of writing,^5^ neither the NATO Strategic Concept (2022) nor the (NATO, 2021) formally recognizes the cognitive domain as a distinct military domain.

### 4.3 (RQ3) The purpose of cognitive warfare

The most inclusive ideal-typical definition of purpose (~90%), “Influencing Behavior,” stipulates that cognitive warfare aims to change how “targets perceive, evaluate, and decide so their actions align with the actor's aims—gaining tactical, operational, or strategic advantage without (or before) kinetic force” (see Table 2). This includes statements suggesting that cognitive warfare is “designed to alter perceptions of reality… to affect attitudes and behaviors… to gain an advantage over an opponent” (Menicocci et al., 2024) and that it “aims to disrupt individual or collective cognition to gain a tactical advantage over an adversary” (Deppe and Schaal, 2024).

**Table 2:** Purpose.

Cluster	Coverage	Title	Ideal-type
1	~90%	Influencing behavior	Change how targets perceive, evaluate, and decide so their actions align with the actor's aims—gaining tactical, operational, or strategic advantage without (or before) kinetic force.
2	~65%	Decision disruption	Impair, slow, or misdirect decision cycles—confusing situational awareness, inducing errors, and degrading tempo.
3	~60%	Societal erosion	Erode trust, cohesion, and legitimacy to fracture social resilience and undermine institutions' ability to respond.
4	~45%	Epistemic rewiring	Reframe reality and authority by attacking knowledge systems and reshaping values and identity so preferred narratives become self-sustaining.
5	~40%	Adversary subversion	Trigger self-reinforcing effects so adversaries effectively further the actor's goals from within.

The second ideal-type definition (~65), “Decision Disruption,” is slightly more specific, stating that the purpose is to “impair, slow, or misdirect decision cycles” to confuse situational awareness, induce errors, and degrade decision-making tempo.” Statements from the corpus suggest that cognitive warfare aims at “the disruption of the Observe, Orient, Decide, Act (OODA) loop,” and “to slow an adversary's decision cycle so his decisions are irrelevant at the time of execution” (Dahl, 1996).

The third ideal-type definition of purpose (~60%), “Societal Erosion,” is about eroding “cohesion, and legitimacy to fracture social resilience and undermine institutions' ability to respond.” One example from the corpus stipulates that the “goal of cognitive warfare is not simply to win battles or achieve short-term victories; rather, it aims to reshape society, undermine trust, and destabilize governments over the long term” (Meghraoui and Belkhamza, 2025).

The fourth ideal type (~45%), “Epistemic Rewiring,” states that the purpose is to “reframe reality and authority by attacking knowledge systems and reshaping values and identity so preferred narratives become self-sustaining.” This includes seeing the desired effects of cognitive warfare as shaping public opinion so that it aligns “with the expected outcomes and goals” (Pace and Coelho, 2022), and “destabilizing institutions, especially governments… initially destabilizing epistemic institutions, such as news media organizations and universities” (Miller, 2023).

The final purpose (~40%), “Adversary Subversion,” is to “trigger self-reinforcing effects so adversaries effectively further the actor's goals from within.” One example states that, rather “than to “compel our enemy to do our will,” the goal is to get the enemy to destroy himself from within, rendering him unable to resist, deter, or deflect our goals” (Bernal et al., 2020).

In essence, the purpose of cognitive warfare is described as influencing behavior by undermining or indirectly steering an adversary's decision space across military, political, and social contexts, ideally achieving strategic effects without kinetic action. While some treat it as a military instrument aimed at shaping the battlespace, others frame it as a broader societal instrument concerned with institutions, public trust, and epistemic stability. This slippage between the operational and the societal highlights dissonances in the corpus, from cognitive processes involved in the OODA loop for military decision-making to questions of societal cognition and cohesion.

### 4.4 (RQ4) The components and methods of cognitive warfare

The most inclusive ideal type (~90%), “Narrative and Information-Influence Toolkit,” identifies “propaganda, disinformation and misinformation, leaks, and framing” as methods used to shape how “people notice, interpret events, and talk about, across various media and everyday conversation” (see Table 3). Statements from the corpus include arguing that “one of the most influential [tools] is disinformation” (Gergelewicz, 2025), that it “exploits vulnerabilities in information ecosystems to manipulate public opinion through the dissemination of dis- and malinformation (and their political variant—propaganda)” (Putter, 2024), and that it uses “disinformation, fake news, propaganda and alternative facts” (Burke and Henschke, 2023).

**Table 3:** Components and methods.

Cluster	Coverage	Title	Ideal-type
1	~90%	Narrative and Information-influence	Using narratives and information tactics—such as propaganda, disinformation and misinformation, leaks, and framing—to steer what people notice, how they interpret events, and how they talk about them, across various media and everyday conversation.
2	~75%	Digital Tech-driven Amplification	Digital platforms and technical systems that produce, tailor, and massively scale influence—botnets, algorithmic amplification, micro-targeting from big data, synthetic media (deepfakes), computational propaganda, and tactical hacks or data leaks.
3	~65%	Exploiting Cognitive Mechanisms	Tactical exploitation of cognitive mechanisms—heuristics and biases, emotion induction, attention saturation, priming, nudges, deception, and timing—to disrupt sense-making and decision cycles.
4	~55%	Coordinated and Covert Societal Engineering	Coordinated and covert campaigns that synchronize information, use psychological and cyber instruments, tailor messages to political, religious, and cultural identities, and mobilize publics as vectors.
5	~40%	Weaponization of the Neuro- and Cognitive Sciences	The deliberate use of findings from neuroscience and cognitive science to shape how people perceive, think, and decide. It includes methods such as designing digital interfaces aligned with human neurocognitive patterns, and, in more speculative forms, employing brain–computer interfaces or neuromodulation to induce specific cognitive responses.

The second ideal type (~75%), “Digital Tech-Driven Amplification,” focuses on “digital platforms and technical systems that produce, tailor, and massively scale influence” and lists examples such as botnets, algorithmic amplification, synthetic media, and computational propaganda. For example, in the corpus, it is stated that cognitive warfare tactics “include cyber elements such as hacking, data manipulation, and the use of social media platforms to spread disinformation” (Karami, 2024) and that “AI-enabled cognitive warfare tactics” include “disinformation and deepfakes” (Fenstermacher et al., 2023).

The third ideal type (~65%), “Exploiting Cognitive Mechanisms,” sees the “tactical exploitation of cognitive mechanisms” as central to “disrupt sense-making and decision cycles.” This involves exploiting heuristics and cognitive biases, emotions, and attention through tactics such as priming, nudging, deception, and timing. Examples include the exploitation of the innate vulnerabilities of the human” (Du Cluzel, 2021) and to create “cognitive overload and thus errors in perception, comprehension, and projection” (Yun and Kim, 2022).

The fourth type (~55%), “Coordinated and Covert Social Engineering,” has a socio-psychological perspective where “coordinated and covert campaigns” are used to “synchronize information, use psychological and cyber instruments, tailor messages to political, religious, and cultural identities, and mobilize publics as vectors.” One statement from the corpus suggests that cognitive warfare “through social engineering can create false alternative realities in democratic states, favored by their freedoms (freedom of speech or free communication of ideas)” (Ionescu and Răpan, 2022) and another that “the use of history, culture, worldview, religion, language, science, and education is of great importance,” as these are essential attack vectors (Danyk and Briggs, 2023).

The final ideal type (~55%), “Weaponization of the Neuro- and Cognitive Sciences,” is specifically focused on leveraging scientific insights to “shape how people perceive, think, and decide.” This ranges from “designing digital interfaces aligned with neuro-cognitive patterns” and “in more speculative forms, employing brain–computer interfaces or neuromodulation to induce specific cognitive responses.” One example of this approach refers to “hacking neuroprosthetic devices to surveil or influence brain activity, or using microorganism-based and other molecular tools to influence cognitive processes” (Ask et al., 2023). Another example discusses “increasingly sophisticated psychological techniques of manipulation (and, potentially, neurophysiological techniques, such as transcranial direct cranial stimulation)” (Miller, 2023).

Regarding methods, there is a broad consensus that cognitive warfare involves the use of manipulated information and propaganda, amplified by computational means. The methods are often described as tailored to exploit heuristic and cognitive biases, which, to a lesser degree, are taken to include the exploitation of social identity. While a non-negligible number of publications emphasize new and emerging methods enabled by cognitive psychology and cognitive neuroscience, few go beyond stating this as a matter of fact. This gives the impression of speculative methods, where the viability and impact at the individual level vs. the societal level are left unanswered.

### 4.5 (RQ5) The distinctions of cognitive warfare

In the corpus, the conceptual domain of cognitive warfare is presented mainly as information and psychological warfare; propaganda and disinformation; and cyber and hybrid warfare (Table 4). However, 19 sources make no distinctions at all.

**Table 4:** Distinctions.

Cluster	Coverage	Title	Ideal-type
1	~80%	Cognition over content	Cognitive warfare leverages information processing—attention, emotion, reasoning—rather than what information the target audiences receive or how it circulates. Where information warfare contests messages and information flows, cognitive warfare targets the mechanisms of perception and decision-making themselves.
2	~55%	Neuro-technological leverage	Cognitive warfare leverages neuroscience and emerging technologies—AI, data analytics, bio- and neuro-inspired methods—to exploit cognitive vulnerabilities. It differs from cyber or psychological warfare by using technology not as a channel but as a means to modulate mental states and decision patterns.
3	~50%	Long-horizon conflict	Cognitive warfare extends conflict into the civilian sphere and across long time horizons. Unlike bounded psychological operations campaigns, it aims at slow, cumulative transformation of public belief, cohesion, and trust through deniable, gray-zone operations.
4	~45%	Cognitive-centric integration	Cognitive warfare integrates psychological, informational, and hybrid tools around a single center of gravity: cognition. It reorders these adjacent forms of warfare so that influencing how people think becomes the strategic principle rather than a supporting function.
5	~35%	Mind as a domain	Cognitive warfare treats the human mind as an operational domain in its own right. Where cyber and electronic warfare target systems and signals, cognitive warfare seeks non-kinetic dominance through perception, interpretation, and will.

The first ideal type (~80%), “Cognition over Content,” sets cognitive warfare in contrast to information warfare, suggesting that the latter focuses on “messages and information flows” while cognitive warfare “attacks the mechanisms of perception and decision-making themselves.” Statements from corpus include presentations of cognitive warfare as “a new phenomenon that differs from any forms of action previously used in the information space” (Drmotová and Kutěj, 2024), that instead of focusing on information, cognitive warfare “has a more subtle yet potentially more damaging goal of shaping not simply what people think, but how they think and how they react to information” (Burke and Henschke, 2023), and “that cognitive warfare stresses less on the direct transmission of messages and more on indirectly influencing the way people absorb knowledge and grow in their beliefs” (Gîndilă, 2024).

The second ideal type (~55%), “Neuro-Technological Leverage,” highlights that cognitive warfare “differs from cyber or psychological warfare by using technology not as a channel but as a means to modulate mental states and decision patterns.” This approach includes statements suggesting that “only cognitive warfare is specifically dedicated to brain control by incorporating weaponized neuroscience into various practices” (Hung and Hung, 2022) and that cognitive warfare transcends “the traditional boundaries of informational and psychological warfare” by combining “modern technologies and knowledge from neuroscience, psychology, and behavioral sciences” (Gîndilă, 2024).

The third ideal type (~50%), “Long-Horizon Conflict,” stipulates that cognitive warfare differs from psychological operations campaigns in that “it aims at slow, cumulative transformation of public belief, cohesion, and trust through deniable, gray-zone operations,” targeting both military and civilian audiences and institutions. One example emphasizes the importance of recognizing “the existence of influence operations that are carried out by non-military actors… aiming to achieve political objectives or even long-term cultural changes” (Ask et al., 2023). Another suggests that cognitive warfare “emerges as a long-term geopolitical strategy that aims to influence the political sphere and generate lasting impacts on society,” and is presented as distinct from psychological operations (da Silva et al., 2025).

The fourth ideal type (~45%), “Cognitive-centric Integration,” views cognitive warfare as an integration of “psychological, informational, and hybrid tools,” in which influencing cognition “becomes the strategic principle rather than a supporting function.” For example, cognitive warfare “encompasses psychological warfare and information warfare but transcends all of them” (Yun and Kim, 2022) and, unlike “traditional cyberattacks or military confrontations, cognitive warfare operates subtly, influencing public opinion, political decision-making, and social stability without the use of overt force or physical combat” (Meghraoui and Belkhamza, 2025).

The final ideal type (~35%), “Mind as a Domain,” treats cognitive warfare as a distinct form of warfare from cyber and electronic warfare, where the latter “target systems and signals,” and cognitive warfare “seeks non-kinetic dominance through perception, interpretation, and will.” In a discussion of the differences between cyber and cognitive warfare, one publication states that the latter “is broader than what is immediately apparent: for example, a cyberattack launched in order… to gain a monetary advantage would not be cognitive warfare; but if the aim is to instill doubt in customers' minds about the bank's ability to be secure, and the perpetrator communicates about this to discredit it, this could be an example of cognitive warfare” (Morelle et al., 2023).

To conclude, at the most general level, cognitive warfare is distinguished from its conceptual predecessors by focusing on human information processing rather than on informational content. The publications engaging with emerging technology and scientific advances highlight that technology is not merely a channel for information, but a means to affect cognition itself. It is, moreover, taken by some to extend conflict into civilian and societal spaces, where long-term influencing of how people think becomes the central strategic principle. Yet significant dissonances remain: the concept slides between efforts to alter cognitive functions and the instrumental use of cognitive psychology and neuroscience to exploit biases and heuristics. Its all-inclusive scope blurs distinctions among existing forms of non-kinetic warfare, raising questions about whether cognitive warfare represents an integration of earlier approaches or offers anything substantively new to the conceptual landscape.

## 5 Discussion

To assess which future avenues of research are needed to enhance the conceptual clarity of cognitive warfare (RQ6), it is first necessary to critically discuss the review. The discussion is normative and begins by engaging with the overall conceptualization of cognitive warfare (RQ1–5), focusing on the most critical sub-concepts, such as cognition and warfare. This is followed by a discussion of the role of neuroscience within the field of research and the concept's potential novelty and utility.

### 5.1 Cognition and warfare

Cognitive psychology and neuroscience encompass a wide range of concepts and constructs that together form a complex framework for understanding human thought, behavior, and the neural mechanisms that underlie them. These disciplines not only examine core cognitive processes but also inform a variety of subfields addressing more specialized phenomena. Within this hierarchical structure, higher- and lower-order functions can be selectively influenced or disrupted through physical, invasive, and non-invasive interventions and technologies. Given this complexity, it is crucial to distinguish clearly between concepts, constructs, and functional levels to maintain analytical precision.

At its core, cognition is here understood to involve several fundamental processes, such as perception (visual, auditory, spatial), language (speech production and comprehension), thinking and reasoning (including decision-making and self-monitoring), memory (working memory, encoding, retrieval), and attention (the ability to focus and manipulate information) (Anderson, 2010; Baddeley, 2007; Piaget, 1952). Moreover, executive functions, responsible for planning, inhibiting impulses, switching between tasks, and working memory updating, oversee and coordinate these underlying cognitive processes (Anderson, 2010; Ochsner and Gross, 2005), making them viable attack vectors because they are essential to how humans operate and make decisions. Further, beyond these internal constructs, cognitive psychology is involved in the explanation of phenomena in other psychological subdisciplines, such as biology (Festinger, 1957; Kandel et al., 2013), social psychology (Neisser, 2014), economics (Tversky et al., 1982), psychometry, ergonomics (Gaines and Monk, 2015), and physical processes of the brain and nervous system, influenced by genetics, hormones, and neurochemical activity (Kandel et al., 2013).

In the corpus, categories drawn from cognitive psychology and neuroscience are widely employed without sustained engagement with the relevant scientific literature. Consequently, cognitive warfare appears as a concept that is superficially scientific yet conceptually underdefined, spanning interpretations from individual cognition to expansive social processes such as religion and ideology. Regardless of whether the research is military or academic in orientation, greater conceptual clarity, particularly a precise definition of cognition, would help to mitigate confusion and strengthen analytical consistency.

Unclear definitions of cognition combined with scientific and technological lingo moreover lead to unclear distinctions from its conceptual siblings. Statements emphasizing that cognitive warfare is a science-driven method for influence more interested in human responses to information than in information itself, appear as anachronistic. Theorizing about psychological warfare in the 1940s, Paul M. A. Linebarger (1948), for example, states that it

“consists of the application of parts of the science called psychology to the conduct of war,” (p. 25)

“designed to affect the minds and emotions of a given enemy, neutral or friendly foreign group for a specific strategic or tactical purpose.” (p. 39)

Writing more generally about propaganda,

Jacques Ellul (1973) similarly holds that it is not about “mere words” but about inciting action. (p. 23)

Moreover, in the literature on information warfare, disseminating information to an enemy is rarely, if ever, described as an end in itself. Its purpose is typically to alter perception so as to induce action and hamper decision-making (Libicki, 1995; Walz, 1998; Whyte et al., 2021).

Regarding warfare more broadly, and from the perspective of classical military thought, the overarching purposes of cognitive warfare are hardly new. The idea of shaping the adversary's perception and will through non-kinetic means has long been part of strategy (McNeilly, 2015; p. 6). Yet, from a Clausewitzian standpoint, definitional precision matters: if war is “an act of force to compel our enemy to do our will” (von Clausewitz, 1984, p. 66), expanding cognition to include political and ideological spheres risks conflating the purpose of cognitive warfare with the purpose of war itself (Dinstein, 2017; p. 17).

The question then arises whether the term *warfare* presupposes war, or whether it is now being applied to activities outside armed conflict. For actors who perceive themselves as engaged in constant warlike confrontation, cognitive warfare appears logical; for others, using the category of warfare without the condition of war risks obscuring the distinction between competition and conflict. This slippage echoes earlier debates on psychological warfare, in which propaganda was considered a constant of political life, and psychological warfare, in conjunction with military, economic, and political means, was seen as its application in the context of war (Qualter, 1962; p. 103).

Moreover, by treating cognition as a separate domain of operations, detached from its psychological and social grounding, cognitive warfare risks becoming superficially scientific yet conceptually hollow (Musholt, 2017, p. 308). While influencing cognition has always been part of strategy, elevating it to an independent domain stretches the concept of warfare beyond its empirical and legal boundaries. As with earlier terminological fads such as hybrid or gray-zone warfare (Azad et al., 2023; p. 83), the danger lies not in recognizing new methods but in eroding conceptual clarity by treating all influence as war. If every form of influence is labeled cognitive, the term loses its analytical and legal coherence: if everything is war, nothing is.

### 5.2 Novelty and utility

In assessing the novelty of cognitive warfare, the concept appears first and foremost to bring together various forms of influence and manipulation that, when cognition is stretched to include general psychological and broader social aspects of human life, have not had a common conceptual frame. This is especially the case when it comes to neuroscience.

The examined literature in this review addresses the role of neuroscience in cognitive warfare primarily from the perspective of cognitive neuroscience, such as heuristics and biases, while other avenues of neuroscience receive notably less attention.

Within the examined body of work, where neuroscience is explored beyond heuristics and cognitive bias, descriptions remain mainly theoretical and lack exemplification. The terms *Neuroweapons, NBIC* (Nanotechnology, Biotechnology, Information technology and Cognitive science) and *Neurotechnology* are mentioned in several texts but lack examples of operationalization, and in many cases even definitions [see (Ask et al., 2023) and (Du Cluzel, 2021) for exemptions and (Danet, 2023) for a critical perspective]. However, these topics have been described more thoroughly under other conceptual frameworks.

While the question of how discourse concerning neuroscience in a national security setting has evolved over the years warrants detailed examination in its own right, a comprehensive review falls outside the scope of this article.

The idea of emerging technologies (Kosal, 2020; Urbina et al., 2022) and neuroscience being used in hostile applications is not new (Krishnan, 2016; Moreno, 2012; Tracey and Flower, 2014), and has been examined through various conceptual lenses throughout the years. In bioethics, potential dual-use of neuroscience and neurotechnology (sometimes referred to as *neuro S/T*) is explored extensively within the field of neuroethics (Giordano, 2014; Dando, 2020).

Potential military applications of neuroscience and neurotechnology have been examined across various contexts, including military medicine, enhancement technologies, and the use of *neuroweapons* (Dando, 2015; DeFranco et al., 2020; Giordano, 2014; Ienca et al., 2018). Although exemplifications of specific technologies encompassed by the term *neuroweapons* vary, commonly cited examples include chemical and biological agents, as well as nano- and biotechnologies (McCreight, 2014). Neuroweapons have been defined as being “intended to influence, direct, weaken, suppress, or neutralize human thought, brainwave functions, perception, interpretation, and behavior”. However, research centering around neuroweapons has generally not situated them within the framework of cognitive warfare.

While this is a limited discussion focusing primarily on cognitive psychology and neuroscience, what is new with cognitive warfare lays less in conceptual understanding than in the scientific application, methods, and technologies through which influence is pursued. Similar to how the concept of hybrid warfare entered Western military thought, cognitive warfare's prime utility might thus be as a political and bureaucratic tool to raise awareness, drive capability development, and secure resources in a world where holistic, multidomain, and transdisciplinary research are a necessity to understand and mitigate an increasingly complex threat landscape.

### 5.3 Future research

Turning to future research for both policy-oriented and academic purposes, it is clear that it should be properly rooted in the earlier literature on propaganda, psychological warfare, and information warfare, and pursued through transdisciplinary collaboration. From a policy perspective, the aim is to avoid expending scarce time and resources on recirculating familiar debates, chasing intellectual fads, or becoming entangled in conceptual detours that impede practical progress. From an academic perspective, the aim is likewise to avoid anachronistic presentism while foregrounding the richness of historical scholarship and recovering earlier insights that can be revisited and tested in light of contemporary scientific knowledge. Across both policy-oriented and academic domains, there is much to be gained from a more informed understanding of one another's conceptual logics and practical constraints.

At this stage in the field's development, three areas of research stand out as particularly important. The first is conceptual clarity. This pertains not only to cognitive warfare as a concept but also, and importantly, its sub-concepts (e.g., cognition and warfare). Clarifications about the scope of cognitive warfare are also necessary: Is it about specific operational theaters or society at large? What does the scope of cognitive warfare imply for our understanding of it? Is warfare always a valid descriptor? If so, what are the ethical, legal, and political consequences?

Second, cognitive warfare continues to suffer from a lack of rigorous operationalization, whether understood as an operational tool or an analytical concept. More empirical work is needed to evaluate documented cases and plausible future effects, and to ensure scientific discipline that avoids speculation and threat inflation. Importantly, treating cognition as more than an extension of information operations suggests new research pathways, particularly the study of how executive cognitive functions might be targeted through invasive and non-invasive neurological, biological, or electronic techniques, and, crucially, the development of effective strategies to mitigate and protect against such threats.

Third, when examining cognitive warfare from an adversary's perspective, it is essential to understand how they conceptualize, operationalize, and legitimize what *we* designate as cognitive warfare. This includes attending to the intellectual traditions, strategic cultures, and doctrinal frameworks through which such activities are rendered meaningful and actionable. Crucially, any such analysis must avoid mirror-imaging, for example projecting *our* own rationality, assumptions, and worldview onto the other, which risks distorting both their intentions and their operational logic, potentially leading to misdirected analysis and mitigation measures.

## 6 Conclusion

This review examines how cognitive warfare is conceptualized in the emerging literature, using a systematic methodology supported by LLM-assisted analysis to identify both converging and diverging understandings. The findings show that the field remains in an early stage of development, with broad conceptual claims often outpacing empirical grounding and scientific rigor. Although many elements of cognitive warfare appear to be a reframing of long-standing practices, the concept nonetheless plays a political and analytical role by directing renewed attention to enduring modes of influence and conflict that have received limited focus in recent years.

Taken together, these insights suggest several priorities for future research. Greater conceptual clarity is needed to delineate what distinguishes cognitive warfare from related concepts, and more robust empirical operationalizations are required to test assumptions and evaluate effects. Moreover, a deeper engagement with how adversaries define, legitimize, and operationalize cognitive warfare is essential to avoid mirror-imaging and improve analytical accuracy. Advancing the field will depend on sustained interdisciplinary collaboration that integrates cognitive science, strategic studies, psychology, political science, and related domains.

## Data Availability

The raw data supporting the conclusions of this article will be made available by the authors, without undue reservation.

## Footnotes

1 In a later edition of the book, Gerring (2012) revises the list to include seven criteria: resonance, domain, consistency, fecundity, differentiation, causal utility and, operationalization.

2 Sources in Korean, Farsi, Arabic, Russian, and Ukrainian were excluded during the screening process.

3 In more detail, the LLM workflow used OpenAI's GPT-4-Turbo (May 2024) together with the text-embedding-three-large model. Each definitional excerpt was vectorized into a 3,072-dimensional embedding representing its latent semantic structure. Pairwise similarity was then computed using cosine similarity (NumPy implementation), and excerpts with similarity scores ≥0.82 were grouped into the same conceptual cluster. GPT-Four-Turbo, guided by controlled synthesis prompts, was subsequently used to generate the ideal-typical representations.

4 To classify academic fields, the ISCED Fields of Education and Training classification (ISCED-F 2013) was used.

5 In the NATO Warfighting Capstone Concept, cognitive superiority is presented as one of six pillars.
