# Supplementary material for: Definitional ambiguity in cognitive warfare: a critical and systematic conceptual review through ideal-type analysis
Source: Front Big Data. 2026 May 15;9:1762571. doi: 10.3389/fdata.2026.1762571 (PMC13218914; doi:10.3389/fdata.2026.1762571)
Supplement: Supplementary file 1 [file Supplementary_file_1.docx]

# Appendices

## Appendix 1: Corpus

1. Ask, Torvald F., Ricardo Lugo, Stefan Sütterlin, Matthew Canham, Daniel Hermansen, and Benjamin J. Knox. “The UnCODE System: A Neurocentric Systems Approach for Classifying the Goals and Methods of Cognitive Warfare.” Preprint, PsyArXiv, 18 May 2023. <https://doi.org/10.31234/osf.io/7c2ez>.
2. Bachmann, Sascha Dov, and Tdhj Org. “Hamas–Israel: TikTok and the Relevance of the Cognitive Warfare Domain.” *The Defence Horizon Journal*, 14 March 2024. <https://tdhj.org/blog/post/hamas-israel-tiktok-war/>.
3. Backes, Oliver, and Andreas Swab. *Cognitive Warfare: The Russian Threat to Election Integrity of the Baltic States*. Policy Analysis Exercise. Harvard Kennedy School: Belfer Center for Science and International Affairs, 2019. <https://www.belfercenter.org/publication/cognitive-warfare-russian-threat-election-integrity-baltic-states>.
4. Bernal, Alonso, Cameron Carter, Kathy Cao, and Olivia Madreperla. *Cognitive Warfare: An Attack on Truth and Thought*. North Atlantic Treaty Organisation (NATO), John Hopkins University, 2020. <https://www.innovationhub-act.org/sites/default/files/2021-03/Cognitive%20Warfare.pdf>.
5. Burke, Paul, and Adam Henschke. “I Know My Truth... Now Tell Me Yours: From Active Measures to Cognitive Warfare in the Russian Invasion of Ukraine.” *Strategic Panorama*, no. 2 (August 2023): 12–27. <https://doi.org/10.53679/2616-9460.2.2022.02>.
6. Chiriac, Olga. “Military Applications of the Cognitive Sciences: Cognitive Warfare, a Matter of Perception and Misperception.” *International Scientific Conference: Strategies XXI* 18, no. 1 (2022): 474–84. <https://doi.org/10.53679/2616-9460.2.2022.02>.
7. Chiriac, Olga R. “Cognitive Warfare in the 21st Century Great Power Competition—Framing of Military Activity in the Black Sea .” *Romanian Military Thinking*, Romanian Military Thinking, 2021, 54–71.
8. Claverie, Bernard. “What Is Cognition? And How to Make It One of the Ways of the War?’ In *Cognitive Warfare: The Future of Cognitive Dominance*, edited by Bernard Claverie, Baptiste Prébot, Norbou Buchler, and François Du Cluzel. NATO Collaboration Support Oﬀice, 2022.
9. Claverie, Bernard, and François Du Cluzel. “‘Cognitive Warfare’: The Advent of the Concept of ‘Cognitics’ in the Field of Warfare.” In *Cognitive Warfare: The Future of Cognitive Dominance*, edited by Bernard Claverie, Baptiste Prébot, Norbou Buchler, and François du Cluzel. NATO Collaboration Support Office, 2022. <https://hal.archives-ouvertes.fr/hal-03635889>.
10. Dahl, Arden B. *Command Dysfunction: Minding the Cognitive War*. Thesis, School of Advanced Airpower Studies, Air University, 1996. <https://doi.org/10.21236/ADA360756>.
11. Danet, Didier. “Cognitive Security: Facing Cognitive Operations in Hybrid Warfare1.” *European Conference on Cyber Warfare and Security* 22, no. 1 (2023): 161–68. <https://doi.org/10.34190/eccws.22.1.1442>.
12. Danyk, Yuriy, and Chad Briggs. “Modern Cognitive Operations and Hybrid Warfare.” *Journal of Strategic Security* 16, no. 1 (2023): 35–50. <https://doi.org/10.5038/1944-0472.16.1.2032>.
13. Deppe, Christoph. “Disinformation In Cognitive Warfare, FIMI, Hybrid Threats.” *The Defence Horizon Journal*, October 16 (2023): online.
14. Deppe, Christoph, Alexandru Fotescu, and Dr Gary S Schaal. *The Understanding of Cognitive Warfare in Comparative Perspective: Taking Stock and Bridging the Gap to Extant Literatures*. NATO Collaboration Support Office, 2024, P13:1–24.
15. Deppe, Christoph, and Gary S. Schaal. “Cognitive Warfare: A Conceptual Analysis of the NATO ACT Cognitive Warfare Exploratory Concept.” *Frontiers in Big Data* 7 (November 2024): 1452129. <https://doi.org/10.3389/fdata.2024.1452129>.
16. Drmotová, Kristýna, and Libor Kutěj. “Cognitive Warfare as a New Dimension of Security. A Fictional Concept or a Real Silent Threat?” *Vojenské Rozhledy* 33, no. 1 (2024): 63–83. <https://doi.org/10.3849/2336-2995.33.2024.01.063-083>.
17. Du Cluzel, François. *Cognitive Warfare*. Nato Act Innovation Hub, 2021. <https://innovationhub-act.org/wp-content/uploads/2023/12/20210113_CW-Final-v2-.pdf>.
18. Fenstermacher, Laurie H., David Uzcha, Kathleen G. Larson, Christine A. Vitiello, and Stephen M. Shellman. “New Perspectives on Cognitive Warfare.” In *Signal Processing, Sensor/Information Fusion, and Target Recognition XXXII*, edited by Lynne L. Grewe, Erik P. Blasch, and Ivan Kadar. SPIE, 2023. <https://doi.org/10.1117/12.2666777>.
19. Ferreira, Vinícius Marques Da Silva, Carlos Alberto Nunes Cosenza, Alfredo Nazareno Pereira Boente, et al. “Cognitive Warfare on Social Networks: Threats, Challenges and Implications for Society.” *ARACÊ* 7, no. 3 (2025): 14287–303. <https://doi.org/10.56238/arev7n3-240>.
20. Gergelewicz, Tomasz. “Countering Disinformation Concept for Building Social Resilience in Times of Cognitive Warfare.” *Przegląd Nauk o Obronności*, no. 20 (February 2025): 23–36. <https://doi.org/10.37055/pno/200300>.
21. Gîndilă, Florin-Marius. “Manipulating Perceptions and Behavior Through Cognitive Warfare Techniques in the Digital Age.” *STRATEGIES XXI—National Defence College* 2, no. 1 (2024): 73–78. <https://doi.org/10.53477/2784-2487-24-08>.
22. Guyader, Hervé Le. “Cognitive Domain: A Sixth Domain of Operations.” Edited by Bernard Claverie, Baptiste Prébot, Norbou Buchler, and François Du Cluzel. Vol. 2021. NATO Collaboration Support Oﬀice, 2022. <https://hal.science/hal-03635898v1/document>.
23. Horowitz, Leonard George. “Trolling for Media Bias, Censorship, and Cognitive Warfare Engineering Psycho-Social Discord.” *Acta Scientific Medical Sciences*, 1 June 2024, 57–67. <https://doi.org/10.31080/ASMS.2024.08.1824>.
24. Hung, Tzu-Chieh, and Tzu-Wei Hung. “How China’s Cognitive Warfare Works: A Frontline Perspective of Taiwan’s Anti-Disinformation Wars.” *Journal of Global Security Studies* 7, no. 4 (2022): ogac016. <https://doi.org/10.1093/jogss/ogac016>.
25. Ibrahim, Fabio, Steffen Rhode, and Monika Daseking. “A Systematic Review of Cognitive and Psychological Warfare.” Preprint, Zenodo, 1 December 2023. <https://doi.org/10.5281/ZENODO.10205600>.
26. Ionescu, Claudiu Marius, and Florian Răpan. “Social Engineering—Major Component of Cognitive Warfare.” *Romanian Military Thinking* 2022, no. 3 (2022): 50–59. <https://doi.org/10.55535/RMT.2022.3.03>.
27. Karami, Ali. “The European Union’s Approach to Cognitive Warfare’s Command and Control.” *Journal of Electrical Systems* 20, no. 11s (2024): 2721–34. <https://doi.org/10.52783/jes.7939>.
28. Krawczyk, Paulina, and Jarosław Wiśnicki. “Russia’s Social-Impact Operations in the Context of Cognitive Warfarein Ukraine in 2022.” *Cybersecurity and Law* 9, no. 1 (2023): 194–203. <https://doi.org/10.35467/cal/169315>.
29. Maksymenko, Serhii, and Lidiia Derkach. “Modern Cognitive and Informational Warfare: The Role of Consciousness Manipulation of Ukrainians in Personality Transformation.” *Collection of Research Papers “Problems of Modern Psychology”*. 64 (November 2024): 157–81. <https://doi.org/10.32626/2227-6246.2024-64.157-181>.
30. Maksymenko, Serhii Dmytrovich, and Lidiya Mykolayevna Derkach. “Understanding Modern Cognitive War in the Global Dimension, Its Genesis in the Ukrainian Context: Cognitive Warfare and Social Impact Operations.” *Obrana a Strategie (Defence and Strategy)* 23, no. 1 (2023): 126–48. <https://doi.org/10.3849/1802-7199.23.2023.01.126-148>.
31. Marsili, Marco. “Guerre à la Carte: Cyber, Information, Cognitive Warfare and the Metaverse.” *Applied Cybersecurity & Internet Governance* 2, no. 1 (2023): 105–20. <https://doi.org/10.60097/ACIG/162861>.
32. Me, Gianluigi, and Maria Felicita Mucci. “Countering Daesh Cognitive and Cyber Warfare with OSINT and Basic Data Mining Tools.” Vol X (2023): 71–80. <https://doi.org/10.19107/CYBERCON.2023.09>.
33. Meghraoui, Loukmane, and Zakariya Belkhamza. “Cognitive Warfare and Cybersecurity: Strategic Implications for Global Security.” 20, 1 (March 2025): 257–64. <https://doi.org/10.34190/iccws.20.1.3277>.
34. Menicocci, Stefano, Viviana Lupo, Silvia Ferrara, et al. “Fake-News Attitude Evaluation in Terms of Visual Attention and Personality Traits: A Preliminary Study for Mitigating the Cognitive Warfare.” *Behavioral Sciences* 14, no. 11 (2024): 1026. <https://doi.org/10.3390/bs14111026>.
35. Miller, Seumas. “Cognitive Warfare: An Ethical Analysis.” *Ethics and Information Technology* 25, no. 3 (2023): 46. <https://doi.org/10.1007/s10676-023-09717-7>.
36. Morelle, Marie, Cegarra Julien, Damien Marion, and André Jean-Marc. “Towards a Definition of Cognitive Warfare.” 2023, 1–4. <https://hal.science/hal-04328461v1>.
37. Muñoz Plaza, Frida, Marco Antonio Sotelo Monge, and Ordi Gonzalez. “Towards the Definition of Cognitive Warfare and Related Countermeasures: A Systematic Review | Proceedings of the 18th International Conference on Availability, Reliability and Security.” *Article 40*, 2023, 1–7. <https://doi.org/10.1145/3600160.3605080>.
38. Nikoula, Daniel, and Dave Mcmahon. *Cognitive Warfare: Securing Hearts and Minds*. University of Ottawa: Information Integrity Lab, 2024. <https://infolab.uottawa.ca/common/Uploaded%20files/PDI%20files/InfoLab%20-%20Cognitive%20Warfare,%20Securing%20Hearts%20and%20Minds.pdf>.
39. Pace, Rodrigo, and Emilio Coelho. “Information as a Weapon of Mass Disruption: From Information Disorder to Cognitive Warfare.” *Revista Da Escola de Guerra Naval* 28, no. 2 (2022): 707–22. <https://doi.org/10.21544/2359-3075.v28n3.g>.
40. Pripoae-Șerbănescu, Ciprian. “Cognitive Warfare—Beyond Dominance, Manoeuvres and Information; The Battle for the Imagined Future.” *Romanian Military Thinking* 2023, no. 4 (2023): 258–79. <https://doi.org/10.55535/RMT.2023.4.16>.
41. Putter, Dries. “Navigating the Interplay of Cognitive Warfare and Counterintelligence in African Security Strategies: Insights and Case Studies.” *Journal of Policing, Intelligence and Counter Terrorism*, 15 December 2024, 1–20. <https://doi.org/10.1080/18335330.2024.2440873>.
42. Rădulescu, Bogdan-George. “How Should Intelligence Analysis Be Utilized to Counter Cognitive Warfare?’ *Intelligence Info* 2, no. 4 (2023). <https://doi.org/10.58679/ii18231>.
43. Reczkowski, Robert, and Andrzej Lis. “Cognitive Warfare: What Is Our Actual Knowledge and How to Build State Resilience?’ *Bezpieczeństwo. Teoria i Praktyka* 3, no. XLVIII (2022): 51–62. <https://doi.org/10.48269/2451-0718-btip-2022-3-003>.
44. Reding, Dale F., and Bryan Wells. “Cognitive Warfare: NATO, COVID-19 and the Impact of Emerging and Disruptive Technologies.” In *COVID-19 Disinformation: A Multi-National, Whole of Society Perspective*, edited by Ritu Gill and Rebecca Goolsby. Springer International Publishing, 2022. <https://doi.org/10.1007/978-3-030-94825-2_2>.
45. Shay, Shaul. “Between Kiev and Venice: The Cognitive Warfare and the Biennale of Venice.” *Security Science Journal* 3, no. 2 (2022): 101–17. <https://doi.org/10.37458/ssj.3.2.6>.
46. Shifei, Li, Han Binpeng, and Yan Ouyang. “Information Feeding for Cognitive Warfare in the Intelligent Era.” *Psychology and Behavioral Sciences*, ahead of print, 14 June 2023. <https://doi.org/10.11648/j.pbs.20231203.11>.
47. Simonyan, Gurgen. “The Problem of Confrontation of the Cognitive Warfare in Case of Inter-Civilizational Conflicts among Narratives.” *The POLITNOMOS․ Journal of Political and Legal Studies* 2, no. 2 (2023): 14–19. <https://doi.org/10.54503/2953-8165-2023.2(2)-14>.
48. Stoian-Karadeli, Andreea and Daniel-Gabriel Dinu. “Securing the Mind: The Emerging Landscape of Cognitive Warfare.” *International Conference RCIC’23: Redefining Community in Intercultural Context*, Brașov (2023): 26–32.
49. Whiteaker, John, and Sami Valkonen. “Cognitive Warfare: Complexity and Simplicity.” Edited by Bernard Claverie, Baptiste Prébot, Norbou Buchler, and François Du Cluzel. NATO Collaboration Support Office, 2022.
50. Yu, Max Tsung-Chi, and Karl Ho. “COVID and Cognitive Warfare in Taiwan.” *Journal of Asian and African Studies* 58, no. 2 (2023): 249–73.
51. Yun, Minwoo and Eunyoung Kim. “Cyber Cognitive Warfare as an Emerging New War Domain and Its Strategies and Tactics.” *The Korean Journal of Defense Analysis* 34, no. 4 (2022): 603–31. <https://doi.org/10.22883/KJDA.2022.34.4.005>.
52. Ördén, Hedvig. “The Neuropolitical Imaginaries of Cognitive Warfare.” *Security Dialogue* 55, no. 6 (2024): 607–24. <https://doi.org/10.1177/09670106241253527>.

## Appendix 2: LLM-prompt

Task Title: Cluster definitions and construct ideal typical definitions ranked by proximity.

Task Description:

1. You will receive a list of statements [definitions/purposes/components and methods/distinctions] regarding the concept “cognitive warfare” drawn from academic or professional sources.

2. Your task is to: a) Carefully read all the statements (some formatting errors might occur, take that into consideration. Disregard references to sources and pages, e.g., [7] (2)); b) Cluster similar statements into conceptual groups based on shared meaning, emphasis, or nuance; c) Formulate one “ideal typical definition” per group that abstracts and synthesizes the common elements; d) Rank the ideal types by proximity from the broadest and most inclusive ideal type to the narrowest and most specific ideal type; and e) quantify approximate coverage (e.g., “covers ~80% of definitions”) for each ideal type.

3. Instructions: a) Use your own words to synthesize the ideal typical definitions; do not merely copy or paraphrase; b) Sort the resulting ideal types from broadest to narrowest; c) Summarize in a final table showing cluster number, name, short description, and % coverage.

4. Tone: Clear, organized, analytic, and practical.
